# Sustaining a Promising Clinical Practice in High-Turnover Rural Environments Through the Geriatric Referral Navigator Role: Qualitative Case Study

**DOI:** 10.2196/81305

**Published:** 2026-03-25

**Authors:** Eileen M Dryden, Camilla B Pimentel, Jessica Riley, Meaghan A Kennedy, Lauren Moo, Laura M Kernan, Lynn A Garvin, Lynette R Kelley, William Hung

**Affiliations:** 1 VA Bedford Healthcare System Center for Health Optimization and Implementation Research Bedford, MA United States; 2 Department of Population and Quantitative Health Sciences UMass TH Chan School of Medicine Worcester, MA United States; 3 Geriatric Research Education and Clinical Center Bedford, MA United States; 4 Department of Public Health University of Massachusetts Lowell Lowell, MA United States; 5 Boston University Chobanian & Avedisian School of Medicine Boston University Boston, MA United States; 6 Harvard Medical School Harvard University Boston, MA United States; 7 Department of Orthopedics Dartmouth–Hitchcock Medical Center Lebanon, NH United States; 8 Department of Health Law, Policy and Management School of Public Health Boston University Boston, MA United States; 9 VA Eastern Colorado Health Care System Aurora, CO United States; 10 James J. Peters VA Medical Center New York, NY United States; 11 Icahn School of Medicine Mount Sinai Hospital New York, NY United States

**Keywords:** sustainment strategies, navigators, access, telemedicine, rural

## Abstract

**Background:**

Sustaining evidence-based health care programs is challenging, especially in clinical settings with high staff turnover. GRECC Connect is an evidence-based telemedicine service that provides geriatric specialty care to rural patients through a hub-and-spoke model between US Department of Veterans Affairs medical center “hubs” and community-based outpatient clinic (CBOC) “spokes.” Four geographically diverse GRECC Connect hub sites (of 19 total) volunteered to implement a “geriatric referral navigator” role to address the challenge of sustaining use of this program in rural CBOCs.

**Objective:**

This study aimed to understand how a health care program such as GRECC Connect can design and implement a navigator role to sustain use of its services.

**Methods:**

We conducted a longitudinal qualitative study using a case study approach. Participants were GRECC Connect hub site staff, including program directors, coordinators, and analysts from a range of disciplines, including clinical pharmacists, geriatricians, and social workers. Over one year, we conducted 31 qualitative interviews (5 to 9 “periodic reflection” meetings and 2 semistructured interviews at each of the 4 sites) focused on key tasks, skills, and characteristics of the geriatric referral navigator role along with perceptions about the role’s value. Each of the group interviews involved 2 to 4 staff members (N=10). We conducted directed content analysis using a rapid analytic approach and then shared deidentified preliminary findings at a national GRECC Connect online meeting where staff from all 19 hub sites (n=40) reflected on the role. We summarized and compared the perspectives shared during that meeting with the data we collected and analyzed from the 4 participating hub sites.

**Results:**

Key navigator tasks included building relationships, providing education, monitoring and troubleshooting logistical and technological issues within and across CBOCs, and evaluating the appropriateness of referrals. While professional backgrounds varied, navigator traits deemed essential for success included being flexible, creative, and a problem solver with deep institutional knowledge. The time needed to conduct navigator tasks—between 15% and 70% of the time required of a full-time employee—was substantial. The navigator role resembles several roles described in the literature that are meant to support program implementation and service use, including internal champions, external facilitators, and clinical navigators. Navigator tasks reflected a combination of known “semivisible” implementation strategies that hub site staff considered necessary not only for *initially* implementing the program but also for *sustaining* use of GRECC Connect in rural clinical environments with high staff turnover.

**Conclusions:**

The geriatric referral navigator role encompasses a broad array of recognized implementation strategies. It is critical to invest in supporting the types of tasks and strategies implemented by the geriatric referral navigator to maintain promising practices over time, where appropriate, to avoid the costs and burdens of implementing new, similar programs in the future.

## Introduction

Implementation science literature describes numerous strategies to spread and, to a lesser extent, sustain use of evidence-based programs in clinical settings, such as the use of internal champions and external facilitators and the development and distribution of educational materials to support awareness and knowledge of programs [1-4]. However, many promising programs created to address challenges in the health care system are not sustained [5-8]. Inadequate staffing has been identified as a key challenge to achieving sustainment [8-10], which is defined by Shelton et al [11] as “the continued use of program components at sufficient intensity for the sustained achievement of desirable program goals and population outcomes.” There is a call to describe approaches used to achieve sustainment in general [12], and given that the health care system in the United States is currently experiencing a protracted period of staff shortages and turnover since the start of the COVID-19 pandemic [13], there is a distinct need for approaches to support sustainment in environments with unstable staffing.

Programs implemented within the context of the US Department of Veterans Affairs (VA) rural community-based outpatient clinics (CBOCs) are particularly vulnerable to sustainment challenges posed by unstable staffing [14]. The VA’s integrated health care system—the Veterans Health Administration—mandates access to care for all enrolled veterans. A large and growing proportion of the VA’s patient population are older and live in rural areas [15]. Thus, the VA has developed an extensive network of CBOCs and numerous virtual and in-person programs and services to care for this population, only a few of which are on-site at the rural clinics [14,16]. CBOCs are, by definition, generally located in resource-poor areas and often struggle with high staff turnover [14,16]. Rural clinicians, many new at the clinics, find themselves in the position of being the critical link between patients and the myriad off-site services available to them through referrals [14]. Among these services is GRECC Connect, a promising practice that provides virtual geriatric specialty care to rural older adults.

GRECC Connect is a VA Office of Rural Health–funded program developed in 2014 to address the challenge of providing specialty care to older, rural veterans. GRECC Connect uses a telehealth hub-and-spoke model that provides rural patients with access to multidisciplinary geriatrics teams in larger “hub site” medical centers [17-19]. CBOC primary care providers make referrals to GRECC Connect for their older, medically complex patients. The patients, and caregivers as needed, can participate in virtual visits from a specially equipped room in the CBOC via a clinical video telehealth platform or from their home via the VA Video Connect app. While rural CBOC staff, patients, and caregivers value the service [14,20], maintaining awareness, knowledge, and consistent use of the program by its target audience can be incredibly challenging, especially given the high staff turnover in this setting and that several different geriatric consultative services may be available.

To address these challenges, as part of a pilot project, 4 GRECC Connect hub sites implemented a “geriatric referral navigator” role to maintain CBOC staff’s use of GRECC Connect services and improve the appropriateness of referrals received from CBOCs. Given the unique context of VA outpatient clinics, GRECC Connect leadership predetermined that part of the role would involve assisting CBOC clinicians in navigating the complexity of referral options for rural patients. However, each hub site had flexibility to define other aspects of this role and associated strategies aimed at sustaining use of GRECC Connect services within the context of their individual sites. We used a case study approach to understand how a health care program such as GRECC Connect can design and implement a navigator role to promote sustained use of its services.

## Methods

### Study Design

We conducted a longitudinal qualitative study using a case study approach.

### Study Team

The overall evaluation team was called the Qualitative Evaluation Core (QualEC) and consisted of 8 VA researchers with expertise in program evaluation, qualitative methods, implementation science, and geriatric medicine. The subgroup of this team that conducted interviews was led by an anthropologist (ED) with more than 25 years of experience leading qualitative research and evaluation studies and included health service researchers with substantial experience in public health and qualitative methods (LMK, LAG, and JR). The entire QualEC team guided the study design and contributed to the interpretation of the findings.

### Setting and Recruitment

In the spring of 2021, we discussed the geriatric referral navigator role during a national GRECC Connect “all-site monthly forum” online meeting with team members from all 19 hub sites in attendance and asked for volunteers to implement the role at their hub site. We then invited the clinical leads of 4 hub sites to participate in this project based on their expressed interest in implementing the navigator role, the availability of a person able to serve in the role, and the geographic diversity of their sites. All GRECC Connect hub sites work with CBOCs whose patient population is more than 50% rural or highly rural.

### Participants

All GRECC Connect staff from the 4 participating hub sites, including individuals filling the geriatric referral navigator role, were invited via email to participate in group interviews and periodic reflections. The data collection activities were scheduled for times when all members of the team were available to make participation more feasible.

### Data Collection

We met virtually via Microsoft Teams with each of the 4 hub site teams for an initial interview to discuss their vision for the “geriatric referral navigator” role (hereafter referred to as the “navigator role”). We continued to meet with the hub site teams every month or two over the course of the pilot year to collect “periodic reflections” [21]. Periodic reflections are a set of structured questions that ask participants to reflect on an implementation process at numerous time points throughout a project. This method enabled us to capture detailed information about participants’ changing perspectives, decision-making processes, and resulting adaptations to the project over time. At each meeting, the hub site teams were asked to discuss and share the data they were collecting and reflect on current pilot program activities, what was working well, what was challenging, changes in implementation plans, and lessons learned. In addition to these periodic reflections, a final interview was conducted with each hub site team during which they shared their perspectives on the navigator role in general, including essential tasks engaged in, necessary skills or characteristics of a successful navigator, the value of the role, and lessons learned for other sites considering having a navigator. All team members were invited to participate together in each of the data collection activities, allowing them to respond to each other’s reflections, thereby providing a more comprehensive view of the navigator role. Interviews and periodic reflections were audio recorded on Microsoft Teams and transcribed.

Once the periodic reflection and interview data were analyzed, we shared the deidentified findings with the GRECC Connect national team during an online meeting—the all-site monthly forum—in November 2022 and asked participants to reflect on the tasks and characteristics associated with the role, the time dedicated to it, and value of the role. This activity also provided an opportunity for member checking [22], as all team members from hub sites implementing the navigator role participated in this national meeting. The meeting was conducted and recorded on Microsoft Teams while 2 QualEC team members took notes.

### Data Analysis

We conducted a directed content analysis [23] using a rapid analytic approach [24,25] to identify essential navigator tasks, beneficial navigator skills and characteristics, barriers to and facilitators of implementing the navigator role, and the perceived value of the navigator role. These approaches combine rigor with speed and were chosen to allow us to generate credible findings within our project’s time frame. We started with a priori coding categories based primarily on topics of interest that were the focus of the interviews and periodic reflections and included the following: description of hub site staff; site context, including patient geographic catchment area (the area for which each hub site is responsible for providing services); key problems to be addressed by implementing the navigator role; description of the navigator role and associated tasks and activities; implementation challenges; and lessons learned. Emergent categories captured additional content relevant to the sites’ implementation of the navigator role.

Consistent with a rapid approach, 3 QualEC team members (ED, JR, and LG) reviewed the transcripts and systematically organized the data into 4 structured individual site-level templates—one for each GRECC Connect hub site. The team met regularly to develop consensus about how to organize the interview and periodic reflection content among the categories within the template. We then synthesized the information within each category across the site-level templates using constant comparison to create a robust description of the navigator role and its perceived value for supporting ongoing use of the GRECC Connect services at the targeted CBOCs.

Two members of the QualEC team took detailed notes at the GRECC Connect national all-site monthly forum meeting where we shared our preliminary deidentified findings on the navigator role. We then augmented the notes with quotes from the meeting’s Microsoft Teams recording. We summarized and compared the perspectives shared during that meeting with the data we collected and analyzed from the 4 participating hub sites, noting areas of alignment or divergence.

### Ethical Considerations

The need for ethics approval was waived by the VA Bedford Healthcare System Institutional Review Board, which determined this program evaluation activity to be nonresearch. As a nonresearch activity, formal informed consent was not required; however, participants were informed about the project plans, including that participation was voluntary, they could opt out of being recorded, and all data collected would be deidentified for publication purposes. Under VA policy, no compensation is provided to VA staff for their participation in interviews.

## Results

### Hub Site Characteristics

**Figure 1:**
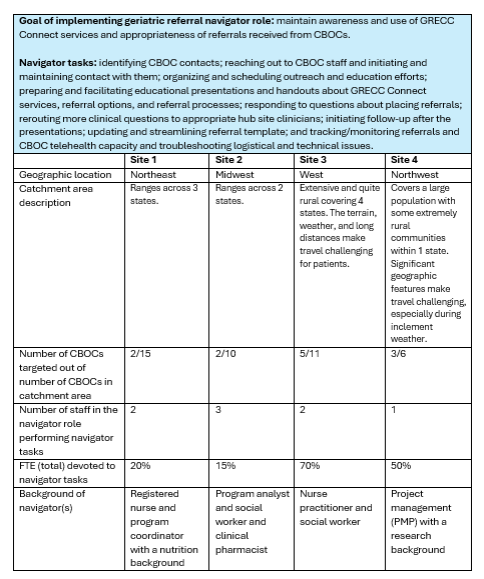
Geriatric Referral Navigator Role cross-site summary.

Each hub site’s catchment area differed in size, terrain, and weather conditions, which may exacerbate health care access issues. Some hub sites worked with patients across state lines, complicating service provision. The number of CBOCs affiliated with each hub site ranged from 6 to 15, and the subset of CBOCs initially working with each navigator ranged from 2 to 5 (Figure 1).

### Participant Characteristics

In total, 2 to 4 staff members from each of the 4 hub sites participated in interviews and periodic reflections (N=10). Participants included GRECC Connect program directors, coordinators, and analysts from a range of disciplines, including clinical pharmacists, geriatricians, licensed nurses, and social workers. Between June 2021 and September 2022, each site participated in 5 to 9 reflection meetings (total of 23 meetings across all sites) and 2 semistructured interviews (a total of 8 interviews across all sites). Reflections and interviews each lasted approximately 60 minutes.

### Navigator Responsibilities

While each hub site was responsible for establishing their own version of the navigator role, they addressed the same barriers: CBOCs’ limited sustained general awareness of GRECC Connect, unfamiliarity with how to make referrals to GRECC Connect, and lack of consistent champions at the CBOCs. Hub sites varied in their approach and structuring of the navigator role, but their vision for the role was the same: to maintain awareness and use of GRECC Connect services and appropriateness of referrals received from CBOCs. The effort dedicated to navigator role tasks at each hub site varied from 15% to 70% of the time required of a full-time employee, spread across 1 to 3 people.

Participants identified several key tasks performed by the navigators. The navigators spent a significant amount of time building relationships with CBOC staff—from identifying CBOC contacts to initiating and maintaining relationships, in some cases trying to cultivate GRECC Connect champions within each CBOC. They conducted educational presentations to CBOC staff on GRECC Connect referral processes, alternative geriatric referral services, and general geriatric care. Tasks related to the presentations included scheduling, facilitation, and follow-up. Other tasks included monitoring CBOC telehealth capacity, troubleshooting technological and logistical issues, and evaluating the appropriateness and completeness of referrals to identify where more education was needed.

Many of the navigator tasks were meant to address confusion regarding placing geriatric referrals. The need for this work was summed up humorously by one of the navigators as she compared clinicians placing referrals to football players engaging in a desperate effort:

[The CBOC clinicians] don’t know which [referral they] should be choosing. So, they kind of, I don’t know. I don’t want to say they throw a Hail Mary, but they kind of pick one and go from there.

To aid the educational process, navigators developed and distributed decision-making tools. These visual aids were meant to assist clinicians in making decisions about the most appropriate referral options for specific types of older patients. Additionally, they streamlined referral templates and processes, distributed marketing flyers, promoted in-service geriatric education opportunities, and responded to questions about referrals.

Hub sites varied in their implementation of each of these activities. For example, some hub site staff members traveled to the CBOCs to conduct educational presentations, whereas others conducted them over online platforms. Additionally, some navigators focused their outreach work solely on the CBOCs, whereas others also presented information to rural community-based organizations with the goal of spreading awareness of the service to community members who may inquire about it from their VA clinicians. Figure 1 provides a comprehensive cross-site summary.

### Characteristics of Successful Navigators

Navigators’ professional backgrounds differed across sites, with some having clinical training and others coming from research backgrounds. However, there was a set of personality characteristics that the hub sites deemed essential for the role that all navigators demonstrated. These traits included flexibility, creativity, perseverance, and being personable and a “problem-solver.” One navigator described outreach to CBOCs as “a marathon, not a sprint” and went on to say the following:

Be diligent and don’t give up if they say this is the right person—just wait. I had a spreadsheet of the contacts that I reached out to...the same people via email, via Teams, and via phone leaving messages.... Don’t be afraid to ask again. If you don’t hear back in a week, 2 weeks, 3 weeks, I just continue to follow up.

They also demonstrated a social savviness in that they were keenly aware of targeting their messages to CBOC staff of a similar professional background or “level” as themselves and harnessing the help of their hub site colleagues from other health care disciplines as needed. Describing this strategy, one navigator said the following:

The telehealth technicians at the CBOCs are a really good way for me to get my foot in the door and then they take it to the supervisory staff and...then we get anywhere from 10 to 50 minutes at a meeting.

Previous VA experience was also critical as institutional knowledge was needed to successfully help others navigate the VA health care system and services.

### Perceived Value of the Role

Regardless of how the navigator role was implemented at each site, staff saw great value in the role because it provided protected time for connecting with rural CBOC staff and clinicians. They noted that most of these tasks had taken place to some extent since the GRECC Connect hub sites were established. However, the tasks had fallen to team members who did not always have the time to complete them as they were additive to their main job. The reality of the CBOC environment, including high staff turnover and a confusing array of referral options, meant that these tasks were never complete; there was an ongoing need to spend substantial amounts of time developing relationships and educating staff on the GRECC Connect service. The purpose of establishing the navigator role was to formalize and highlight this work, to elevate the tasks into a “role” to allow for a more systematic, intentional, and intensive focus on these tasks. Participants across all sites believed that this role, or these tasks spread out among staff members, was critical for sustaining the GRECC Connect service at their site. Uncertainty about consistent hub site staff funding was both a concern and potential barrier to maintaining the navigator role. One navigator reflected on who would do this important work if the navigator role did not exist:

I think that anybody on the team having a connection to the CBOC or traveling there or being willing to be humbled and outreach to people or offer services...I think is key. ...making those connections and showing up.... It’s just...who has the time and ability to do that?

### Reflections on Navigator Role From GRECC Connect National All-Site Monthly Forum

In November 2022, at GRECC Connect’s national monthly forum, we presented a summary of our findings to all staff, including those from hub sites beyond the 4 participating ones, and asked them to reflect on the geriatric referral navigator role. In total, there were 40 participants representing all 19 GRECC Connect hub sites. Participants’ professions included geriatricians, pharmacists, social workers, administrative staff, and data specialists.

The description of the navigator tasks and the characteristics for success strongly resonated with participants. One navigator noted the following:

The common theme [the pilot navigators] were talking about is being ready to put on whatever “hat” that you need to and learn about new tasks or fulfill responsibilities.... Really, anything that needs to be done to improve visibility and make sure things work and flow smoothly.... The path isn't always straightforward.

While almost all hub site staff members participating in the forum endorsed having conducted some of the noted navigator tasks, program coordinators identified the most with the findings. They perceived that a portion of their job, too, was to increase the visibility and reach of the program. This was in addition to the time they spent as program coordinators on administrative reporting duties and connecting with veterans, families, and clinicians; scheduling telehealth appointments; and providing telehealth support before and during visits.

While many navigator tasks were done to some extent by program coordinators and others, the amount of time spent on those tasks was quite different. For example, one navigator noted sending more than 600 emails over the year to 6 CBOCs to create and maintain relationships, schedule educational sessions, and troubleshoot technical issues. Participants noted that all members of hub site staff are allotted insufficient time to create and sustain relationships with CBOCs and that having any member of staff with dedicated time for those tasks can help provide access to services that would otherwise be overlooked. One GRECC Connect hub site director said the following:

As others have agreed, this [the geriatric referral navigator tasks] is the glue, right? This is what makes “everything” go.

## Discussion

### Principal Findings

GRECC Connect was created to provide needed geriatric specialty care services to rural patients that have limited access to them locally [17]. Within CBOC settings with high levels of staff turnover, maintaining awareness of GRECC Connect and knowledge of how to place a referral has proven challenging and threatens the sustainability of this valuable service. The navigator role was developed to address sustainability barriers faced by GRECC Connect in the rural CBOCs by intentionally carving out protected time for one or more staff members to dedicate to relationship building and education. Participants perceived the navigator tasks to be essential for maintaining the use of GRECC Connect services in dynamic CBOC environments and for ensuring that rural patients who need specialty geriatric care are connected with the right services in a timely manner. However, the time needed to implement these navigator tasks was substantial, and the role was most successful when filled by a uniquely skilled individual.

### Geriatric Referral Navigators as Internal Champions and External Facilitators

The tasks performed by GRECC Connect’s geriatric referral navigators, including building relationships, providing education, guiding referrals, monitoring CBOC telehealth capacities, and troubleshooting logistical and technological issues within and across CBOCs, are reflected in a combination of similar roles described in literature. The navigator exists in an interesting position in that it is “internal” to the GRECC Connect program (they are part of the GRECC Connect hub site) but “external” to the rural outpatient clinics that are the source of GRECC Connect referrals and, therefore, the focus of the implementation uptake and sustainment activities. The tasks of the role and characteristics associated with a successful navigator reflect this inside and outside position and align with at least 2 different types of implementation strategies—“site champions” and “external facilitators.”

Miech et al [26] articulate the key characteristics of champions as “people who (1) are internal to an organization; (2) generally have an intrinsic interest and commitment to implementing a change; (3) work diligently and relentlessly to drive implementation forward, even if those efforts receive no formal recognition or compensation; (4) are enthusiastic, dynamic, energetic, personable, and persistent; and (5) have strength of conviction.” Successful champions are also noted to be good communicators, boundary spanners, and skillful at troubleshooting problems and using data to monitor progress, as well as influencing peers as they educate and collaborate with those who are expected to implement the program [26]. These are very similar attributes to traits that were perceived to be essential for successful geriatric referral navigators in our evaluation. Not having consistent champions to work with at the CBOCs is likely a contributing factor as to why the navigators took on this role themselves.

One difference between the navigators and champions is that champions are often frontline employees that can lead by example [27]. Another difference is that this work is the focus of the navigator’s role and they are being compensated and recognized for doing these tasks. In that way, they are like another key actor in the implementation “active change process” [28]: the external facilitator.

External facilitators have many of the same characteristics of champions, including good communication skills. Because external facilitators are situated outside of the local implementation context, a large part of their role is to “build relationships across organizational boundaries and levels to create a supportive environment for the program” [29]. Given their outsider role, they usually take on a more systems-level approach than site champions, leading and managing processes that affect multiple sites within the system while at the same time helping sites tailor the intervention to fit within their clinic environment [29]. Geriatric referral navigators similarly worked to address some of the more systemic-level challenges they saw arising across multiple CBOC sites, such as helping create easier-to-use referral templates and processes. Their higher-level institutional knowledge also helped them troubleshoot in a way that site champions may not feel they have the knowledge or authority to do, such as when working with telehealth leadership to try to ensure availability of telehealth equipment across CBOCs to support their capacity to engage in these services.

### Geriatric Referral Navigators as Clinical or Triage Navigators

The geriatric referral navigator role is also similar to that of patient navigators [30] and clinical navigators [31] in that their goal is to ease the referral process for both clinicians and patients and ensure that patients get to the right services at the right time. While the “patient navigator” role may be more common and familiar, the geriatric referral navigator role is more akin to both “clinical navigators” and “triage navigators” in that it directly supports clinicians navigating the referral landscape. The VA’s Referral Coordination Initiative, currently being implemented for some types of specialty care at a select number of sites, is a type of triage navigation strategy that serves clinicians [32,33]. It uses a “nurse-first” approach, meaning that referrals first go to nurses who assess and then triage them to the most appropriate services to ensure that patients receive the right care at the right time. This approach shifts tasks from specialty care clinicians to nurses with the intent of creating a more efficient process.

### Navigator Tasks Are Aligned With Consolidated Framework for Implementation Research Constructs and Expert Recommendations for Implementing Change Strategies

The Consolidated Framework for Implementation Research (CFIR) [28] is a well-known implementation science framework that describes 5 domains or constructs that influence program implementation. By combining many of the functions of clinical navigators, internal champions, and external facilitators, geriatric referral navigators address several known barriers to program implementation related to the CFIR constructs of “intervention characteristics,” “inner setting,” and “outer setting.” [28]. Specifically, through their educational efforts that include information on other types of geriatric services, they bolster the perceived “relative advantage” of GRECC Connect. By developing and sharing decision trees and working to simplify referral requirements, they reduce the perceived “complexity” of service use. Through building relationships with local staff and monitoring referrals and technical needs, they align their technical assistance and educational content with “local patient needs and resources.” Finally, the ongoing nature of all the navigator tasks helps address challenges related to staff turnover, one of the most significant “structural characteristics” that impede sustainability.

Often discussed alongside the CFIR are the Expert Recommendations for Implementing Change (ERIC) [4] that were developed by a panel of implementation scientists and include 73 strategies for supporting program implementation. Geriatric referral navigator tasks overlap extensively with ERIC strategies related to educational efforts (eg, conducting educational meetings and outreach visits and developing and disseminating educational materials), monitoring (eg, conducting audits, providing feedback, and developing quality monitoring systems), relationship building (eg, building a coalition and identifying champions and early adopters), and external facilitation activities (eg, sharing local knowledge and advocating for a change in equipment and interactive problem-solving) [4]. Furthermore, as noted by Chambers et al [34] in their dynamic sustainability framework, traits identified across the navigators in this case study, including being flexible, creative problem solvers, help the program adapt as needed to support its sustainability over time. Viewed together, this broad expanse of implementation support provided by the geriatric referral navigator may help not only explain but also justify the relatively significant amount of employee effort needed to accomplish the navigator tasks in our case study.

### Navigator Tasks and Semivisible Labor

It is notable that many of the tasks that were a core part of the navigator role were recognized by GRECC Connect hub site staff, including physicians, as tasks they often did in addition to their “official” work. They described these tasks, such as “ongoing relationship building,” as taking a substantial amount of time, yet they were not necessarily recognized as a part of their job. In this way, these types of tasks could be characterized as “semivisible labor.” Semivisible labor is described by Zogas et al [35] as “activities performed by workers that are necessary and that advance an organization’s objectives, but which are often overlooked or devalued by employers, consumers, and other workers.” Therefore, while critical for maintaining use of the valuable GRECC Connect services, they are not officially part of anyone’s job duties. Zogas et al [35] go on to say the following when describing the efforts of clinical pharmacy practitioners to ensure that their services were used: “[the clinical pharmacy practitioners] did report a need to continue using relationship-building strategies because of turnover in leadership, clinicians, and other clinic staff,” and there would be an ongoing “need for continued efforts at maintaining integration given staff and leadership turnover.”

This strongly aligns with our findings and suggests that the types of tasks that were viewed as integral to the navigator role and critical to sustaining the use of GRECC Connect services are not unique to GRECC Connect. However, geriatrics may be more likely to suffer from these challenges; there may be a lack of immediate recognition of the value of geriatric consultative services given that many clinicians may care for older patients regularly and may not have had the opportunity to experience the benefits of geriatric specialty care for their patients. Ongoing educational efforts may be even more essential to support the sustained use of such services. Especially in the current health care environment with staffing turnover and shortages, it is important to acknowledge and make visible these tasks that are essential to sustaining valuable health care programs. Assigning these tasks to a position such as the geriatric referral navigator could improve efficiency by allowing others on the team to practice at the highest level of their training and expertise.

Our paper characterizes the role of the geriatric referral navigator, a unique approach to supporting sustainment in a context about which little has been written [12]. The tasks performed by a geriatric referral navigator are critical in an environment of high staff turnover—a situation experienced in many rural health care settings. However, when funding shifts and external funds are no longer available to support this role, this valuable service could be in danger of ending. The navigator’s tasks may be more readily recognized as essential for the *initial* implementation phase of a program, not as *ongoing* activities in need of continuous dedicated support. In the context of high turnover and complex referral landscapes, the tasks performed by the navigator are needed continuously to sustain valuable services and should be considered an ongoing program cost. More work needs to be done to make these vital tasks visible and provide the necessary resources to fund them.

### Limitations

This description of the navigator role as a strategy to support the sustainment of a promising practice has limitations. First, we have not yet assessed whether implementing this role does maintain or increase referrals for older patients from rural CBOCs over time. Future research will help determine this. Second, the hub sites that implemented this role were not chosen at random. Because, instead, they expressed interest in piloting this role, they likely anticipated some of the value they later ascribed to it.

### Conclusions

Implementing the geriatric referral navigator role was considered by GRECC Connect hub site staff to be an important strategy to sustain service use in dynamic rural CBOC environments. It is critical to invest in such strategies to maintain promising practices over time, where appropriate, to avoid the costs and burdens of implementing new, similar programs in the future.

## Abbreviations

CBOC: community-based outpatient clinic
CFIR: Consolidated Framework for Implementation Research
ERIC: Expert Recommendations for Implementing Change
QualEC: Qualitative Evaluation Core
VA: United States Department of Veterans Affairs

## Data Availability

The datasets generated or analyzed during this study are available from the corresponding author on reasonable request.
